# Facile synthesis of novel porous self-assembling hydrogen-bonding covalent organic polymers and their applications towards fluoroquinolone antibiotics adsorption†

**DOI:** 10.1039/c8ra06806b

**Published:** 2018-10-01

**Authors:** Zhuoran Li, Feifan Xu, Zhi Liu, Chuanyu Qin, Hao Ren, Yangxue Li

**Affiliations:** Key Lab of Groundwater Resources and Environment, Ministry of Education, Jilin University 2519 Jiefang Road Changchun 130021 P. R. China yangxueli@jlu.edu.cn; School of Municipal and Environmental Engineering, Jilin Jianzhu University 5088 Xincheng Street Changchun 130118 P. R. China; State Key Laboratory of Inorganic Synthesis and Preparative Chemistry, College of Chemistry, Jilin University Changchun 130012 P. R. China

## Abstract

A series of porous hydrogen-bonding covalent organic polymers (H_C_OPs) have been synthesized based on three-composite building blocks through a quick and succinct method for fluoroquinolone antibiotics adsorption from aqueous solutions. The porous properties of the H_C_OPs were regulated and controlled by adjusting the lengths of linkers, and the crystallinity and stability were strengthened due to the introduction of hydrogen bonds in H_C_OPs. Taking advantage of the porous properties and π-conjugated phenyl rings, as well as functional –CO–NH– and –COOH groups, H_C_OPs removed organic pollutants from wastewater effectively and showed good reusability. The external adsorption behavior was analyzed using both kinetic analysis and isotherm analysis. The results showed that the adsorption obeys the pseudo-second order kinetic model and follows the Langmuir isotherm model. The obtained maximum adsorption capacity of the four H_C_OPs was arranged in sequence according to the specific surface areas and pore sizes. Furthermore, the internal mechanisms involving perforated porousness, electrostatic interaction, hydrophobic interaction, π–π electron-donor–acceptor (EDA) interaction and hydrogen bonding formation, were investigated in detail. We envisage broadly applying the H_C_OPs in the facile and effective management of environmental pollution.

## Abstract 2

A series of porous hydrogen-bonding covalent organic polymers (H_C_OPs) have been synthesized based on three-composite building blocks through a quick and succinct method for fluoroquinolone antibiotics adsorption from aqueous solutions.

## Graphical abstract

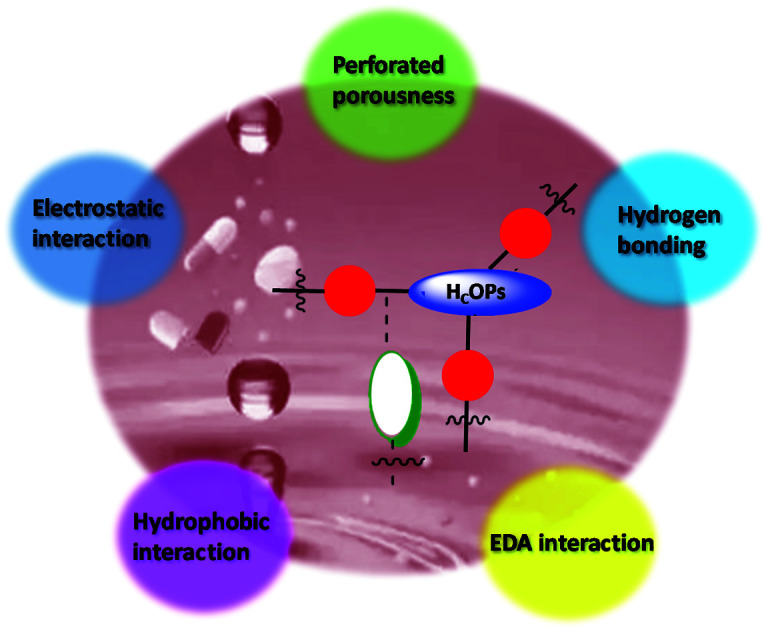

## Introduction

Fluoroquinolones, used in pharmaceutical and personal care products (PPCPs), are synthetic antibiotics widely used in human medical and animal husbandry.^1^ With the improvement of human living standards, such antibiotics are extensively produced and used, and most of them tend to drain into the aquatic environment. Moreover, even a low concentration of antibiotics, will lead to the drug resistance of pathogenic bacteria and pose a serious threat to natural ecosystems and human health. In recent years, different degrees of such antibiotic pollution have been detected in various water environments. For example, in the United States, the concentrations of fluoroquinolone were detected to be 2 and 0.12 μg L^−1^ in municipal wastewater and surface water, respectively;^2^ and in the sewage from German hospitals, the concentration of fluoroquinolone was as high as 124. 5 μg L^−1^.^3^ Xu *et al.* investigated the average concentrations of fluoroquinolone in the mainstream of the Yellow River, 25 to 152 ng L^−1^, whereas the concentrations in the tributaries can reach up to 44 to 240 ng L^−1^.^4^ Therefore, it counts for a great deal to discuss the removal of fluoroquinolones from water. In many antibiotic wastewater treatment technologies, adsorption technology distinguishes itself from others with simple operation, little equipment investment cost and energy consumption, and no production of toxic intermediates.^5^ Considering the defective performance of existing adsorbents, developing new types of adsorbents comes to be burning issues in remediation of wastewater.

On the other hand, covalent organic polymers (COPs) consisted of covalent linkages, including both crystalline and amorphous forms, have been definitely showcased their striking charm across environment domains, healthcare sectors and energy-related fields.^6^ In general, the synthesis of COPs have been realized through employing finite one or two types of monomers to form limited linkages including B–O linkages, C–N linkages, C–C linkages, N–N linkages, *etc.* for constructing 2D/3D motifs.^7^ Apparently, although the progress achieved to what it is today, the restricted species and structure-types of COPs associated with the monotonous building blocks and special bonding modes, making the unsatisfactory full pay of COP values. In this way, exploring novel COPs with multiple linkages and versatile structures is an unquestionable necessity rather than just an option to meet the requirements of promoting application.^8^

Alternatively, hydrogen-bonded organic frameworks (HOFs) which rely on weak interactions *e.g.*, van der Waals force, hydrogen bonding, π–π stacking and so forth, gradually emerge as a new highly innovative subject ever since first proposed by Chen's Group in 2011.^9^ However, the weaknesses of weak stability of HOFs outweigh the strengths of mild synthetic conditions. As a result, the practical applications of HOFs in environmental settings have been obstructed. Inspired by the pioneer work well established by Lin *et al.*, a similar concept could be generated to develop hydrogen-bonded covalent organic polymers (H_C_OPs), which would leverage merits of both COPs and HOFs.^10^

**Scheme 1:**
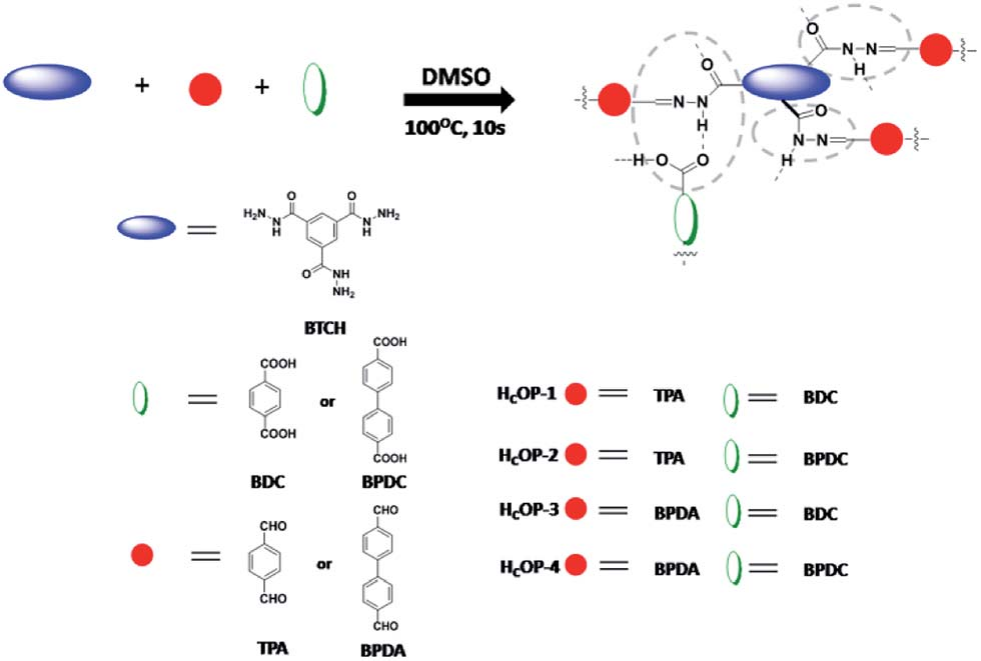
Representative molecular structures of H_C_OPs.

**Scheme 2:**
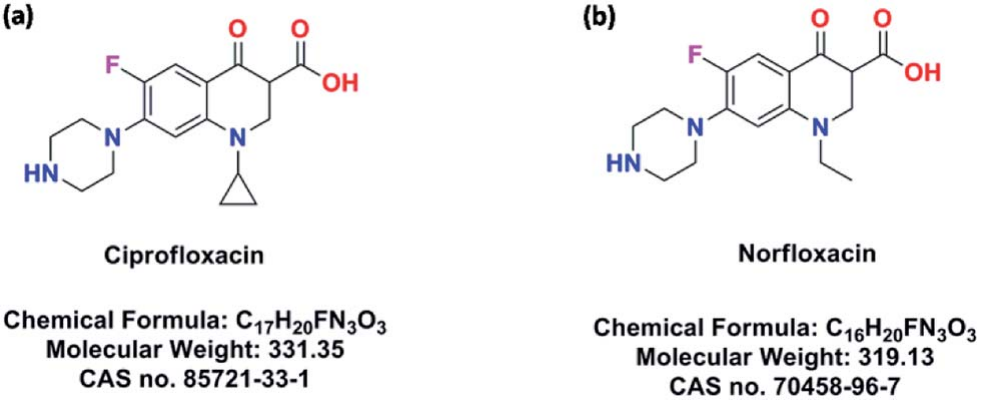
Chemical information of ciprofloxacin (a) and norfloxacin (b).

To evidence this assumption, a series of porous hydrogen-bonding covalent organic polymers (H_C_OPs) were obtained by self-assembly of multiple types of mixed linkers through catalyst-free dynamic imine chemistry and hydrogen bonding within an extremely short time (Scheme 1). The strategy for preparing novel H_C_OPs was designed reasonably: (1) three types of monomers involving hydrazines, aldehydes and carboxylics were chosen as building blocks, bringing new features to the existing porous organic materials; (2) the *C*_3_-symmetric benzene-1,3,5-tricarbohydrazide (BTCH) containing carbohydrazide functional groups, not only could form hydrogen bonds with carboxylic monomers, but also could construct hydrazone linkages with aldehyde monomers; (3) by adjusting the length of linear monomers providing great potential extensibility and diversity of packing patterns; and moreover, (4) the introduction of hydrogen bonding coupled with covalent bonds affording high chemical stability towards selective adsorbing environmental pollutants. Herein, the resulting H_C_OPs were explored as a new type of adsorbents for two typical kinds of fluoroquinolone, *i.e.*, ciprofloxacin (CIP) and norfloxacin (NOR) (Scheme 2). Notably, as far as we know, it was the first-time use of H_C_OPs for adsorbing fluoroquinolone antibiotics to address the environmental issues.

## Experimental

### Reagents and materials

All starting reagents, except benzene-1,3,5-tricarbohydrazide,^7*e*^ were purchased commercially and used directly as received without further purification.

### Characterization

The thermogravimetric analyses (TGA) were performed by heating the H_C_OPs at a heating rate of 10 °C min^−1^ in N_2_ flow. CHN elemental chemical analyses of the H_C_OPs were carried out by an Elementar model Vario Micro analyzer. The fourier transform infrared spectra (FT-IR) of the H_C_OPs were recorded by using a Nicolet Impact 410 Fourier transform infrared spectrometer through a KBr disc method in a range of 400–4000 cm^−1^. The surface areas and porosities of the H_C_OPs were measured on a Micromeritics ASAP 2020 analyzer. The powder X-ray diffraction (PXRD) patterns of the H_C_OPs were conducted by a Riguku D/MAX2550 diffractometer using CuKα radiation with a wavelength of 1.54178 Å. The morphologies and structures of the H_C_OPs were probed by field-scanning electron microscopy (FE-SEM, JEOLJXA-840, 15 kV) and the solid-state ^13^C cross-polarization/magic-angle spinning nuclear magnetic resonance (CP/MAS NMR, Bruker AVANCE III NMR spectrometer, 400 MHz). The UV-vis diffuse reflectance of the H_C_OPs spectra were recorded by a UV-vis spectrophotometer (UV-2550, Shimadzu) at room temperature. Point zero charge of the H_C_OPs were measured at various pH with a JS94H (Shanghai, China).

### Synthesis of H_C_OP-1

A mixture of benzene-1,3,5-tricarbohydrazide (BTCH, 0.2 mmol, 0.05 g), terephthalaldehyde (TPA, 0.3 mmol, 0.04 g) and terephthalic acid (BDC, 0.3 mmol, 0.05 g) in dimethyl sulphoxide (DMSO, 10 mL) was stirred and heated at 100 °C for about 10 s. After that, the resulting yellow polymer (H_C_OP-1) could be achieved with 94% yield. Elemental analysis (wt%) calcd. For {C_28_H_21_N_6_O_7_}_*n*_: C 60.76, H 3.82, N 15.18; found: C 60.55, H 3.97, N 15.74.

### Synthesis of H_C_OP-2

A mixture of benzene-1,3,5-tricarbohydrazide (BTCH, 0.2 mmol, 0.05 g), terephthalaldehyde (TPA, 0.3 mmol, 0.04 g) and biphenyl-4,4′-dicarboxylic acid (BPDC, 0.3 mmol, 0.07 g) in dimethyl sulphoxide (DMSO, 10 mL) was stirred and heated at 100 °C for about 10 s. After that, the resulting yellow polymer (H_C_OP-2) could be achieved with 95% yield. Elemental analysis (wt%) calcd. For {C_34_H_25_N_6_O_7_}_*n*_: C 64.86, H 4.00, N 13.35; found: C 64.55, H 4.16, N 13.74.

### Synthesis of H_C_OP-3

A mixture of benzene-1,3,5-tricarbohydrazide (BTCH, 0.2 mmol, 0.05 g), 4,4′-biphenyldicarboxaldehyde (BPDA, 0.3 mmol, 0.06 g) and terephthalic acid (BDC, 0.3 mmol, 0.05 g) in dimethyl sulphoxide (DMSO, 10 mL) was stirred and heated at 100 °C for about 10 s. After that, the resulting yellow polymer (H_C_OP-3) could be achieved with 90% yield. Elemental analysis (wt%) calcd. For {C_34_H_25_N_6_O_7_}_*n*_: C 64.86, H 4.00, N 13.35; found: C 64.85, H 4.07, N 13.39.

### Synthesis of H_C_OP-4

A mixture of benzene-1,3,5-tricarbohydrazide (BTCH, 0.2 mmol, 0.05 g), 4,4′-biphenyldicarboxaldehyde (BPDA, 0.3 mmol, 0.06 g) and biphenyl-4,4′-dicarboxylic acid (BPDC, 0.3 mmol, 0.07 g) in dimethyl sulphoxide (DMSO, 10 mL) was stirred and heated at 100 °C for about 10 s. After that, the resulting yellow polymer (H_C_OP-4) could be achieved with 92% yield. Elemental analysis (wt%) calcd. For {C_40_H_29_N_6_O_7_}_*n*_: C 68.08, H 4.14, N 11.91; found: C 67.95, H 4.16, N 12.04.

### Adsorption experiments

All adsorption experiments were carried out with batch experiments at ambient temperature in air. In a general procedure, 10 mg H_C_OPs were added into 10 mL solution of fluoroquinolones (2–20 mg L^−1^). Thereafter, the mixture was stirred on a rotating shaker for a certain time at ambient temperature. Then the supernatant was measured by UV-vis spectrometry at wavelength of 277 nm for ciprofloxacin and 278 nm for norfloxacin at various time intervals to calculate the residue amount of fluoroquinolones in the solution.

## Results and discussion

### Characterization of H_C_OPs

**Fig. 1:**
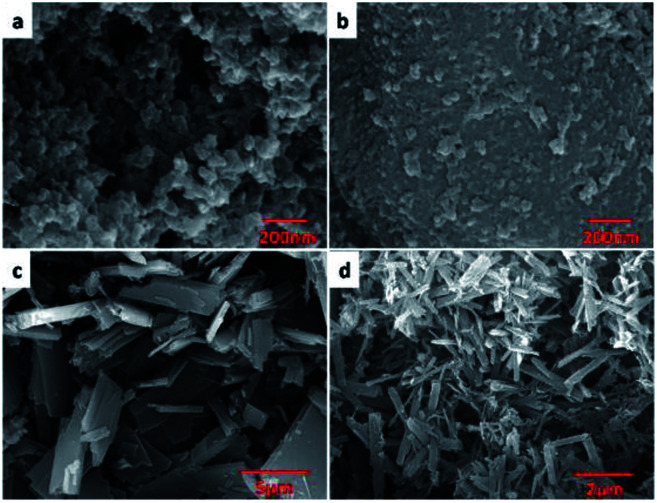
SEM images of H_C_OP-1 (a), H_C_OP-2 (b), H_C_OP-3 (c) and H_C_OP-4 (d).

Scanning electron microscopy (SEM) images showed that H_C_OP-1 and H_C_OP-2 appeared nanoporous interconnected particles, while H_C_OP-3 and H_C_OP-4 exhibited uniform rectangular morphologies, respectively (Fig. 1a–d). The PXRD patterns of the H_C_OPs were in line with the amorphous nature (Fig. S1, ESI†). Interestingly, the sharp peaks were observed in H_C_OPs, corresponding to the hydrogen bonds which could strengthen the crystallinity.^11*a*^ The stability of H_C_OPs was studied in the presence of concentrated HCl. To our surprise, the H_C_OPs didn't dissolve and the PXRD patterns changed little even after 3 days (Fig. S2, ESI†). As we know, the hydrazone linkage is dynamic and acid-sensitive, we suggest that the high stability of H_C_OPs possibly results from the hydrogen bonds formed between the BTCH moieties and BDC (or BPDC) moieties in H_C_OPs.^11*b*^

**Fig. 2:**
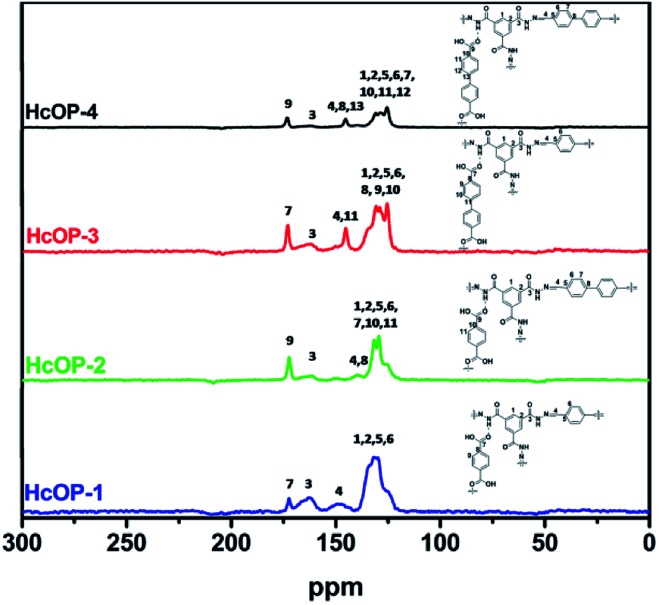
Solid-state ^13^C CP/MAS NMR spectra of H_C_OPs.

The FT-IR spectra of H_C_OPs showed stretching peaks at 1288 cm^−1^ that are characteristic of C=N moieties, implying the occurrence of aldehyde–hydrazine condensation reaction in the H_C_OPs (Fig. S3 and S4, ESI†). Furthermore, when comparing the FT-IR spectra of H_C_OPs to monomers, we found that the stretching vibration of N–H at 3300 cm^−1^ in BTCH blue-shifted towards higher wavelength of 3450 cm^−1^; meanwhile, the C=O stretching band in BDC (or BPDC) red-shifted from 1685 to 1680 cm^−1^. These shifts can be ascribed to hydrogen-bonding interactions between carbonyl and imide.^12*a*^ The successful formation of hydrogen bonds and hydrazone bonds in H_C_OPs were also proved by the ^13^C CP/MAS NMR analysis at the molecular level. As shown in Fig. 2, the characteristic resonances at 170 ppm and 160 ppm of C=O bonds provided the solid evidence for the presence of BDC (or BPDC) and BTCH, respectively. Meantime, the characteristic resonances at 145 ppm of C=N bonds, proving the successful condensation of TPA (or BPDA) and BTCH again.^12*b*^

The H_C_OP-1 and H_C_OP-2 are stable in N_2_ up to 300 °C, while the H_C_OP-3 and H_C_OP-4 are stable in N_2_ up to 350 °C, respectively, as revealed by TGA (Fig. S5, ESI†). The porous structures of the H_C_OPs were investigated by nitrogen sorption measurements at 77 K.^13^ In light of the IUPAC classification, the H_C_OPs all exhibited type IV sorption isotherm curves (Fig. S6 and S7, ESI†). Among the four H_C_OPs, H_C_OP-4 displayed the relatively highest N_2_ uptake with a BET surface area of 43 m^2^ g^−1^ (Table S1, ESI†). And the remaining H_C_OPs were arranged in descending order by the surface areas: H_C_OP-3 (41 m^2^ g^−1^) > H_C_OP-2 (18 m^2^ g^−1^) > H_C_OP-1 (13 m^2^ g^−1^). The Brunauer–Emmett–Teller (BET) surface areas of H_C_OPs increased along with increasing length of linkers, briefly, the surface areas are proportional to the length of linkers under the reaction conditions used. This finding is in accordance with the previous PTPA network.^14^

### Adsorption properties

**Fig. 3:**
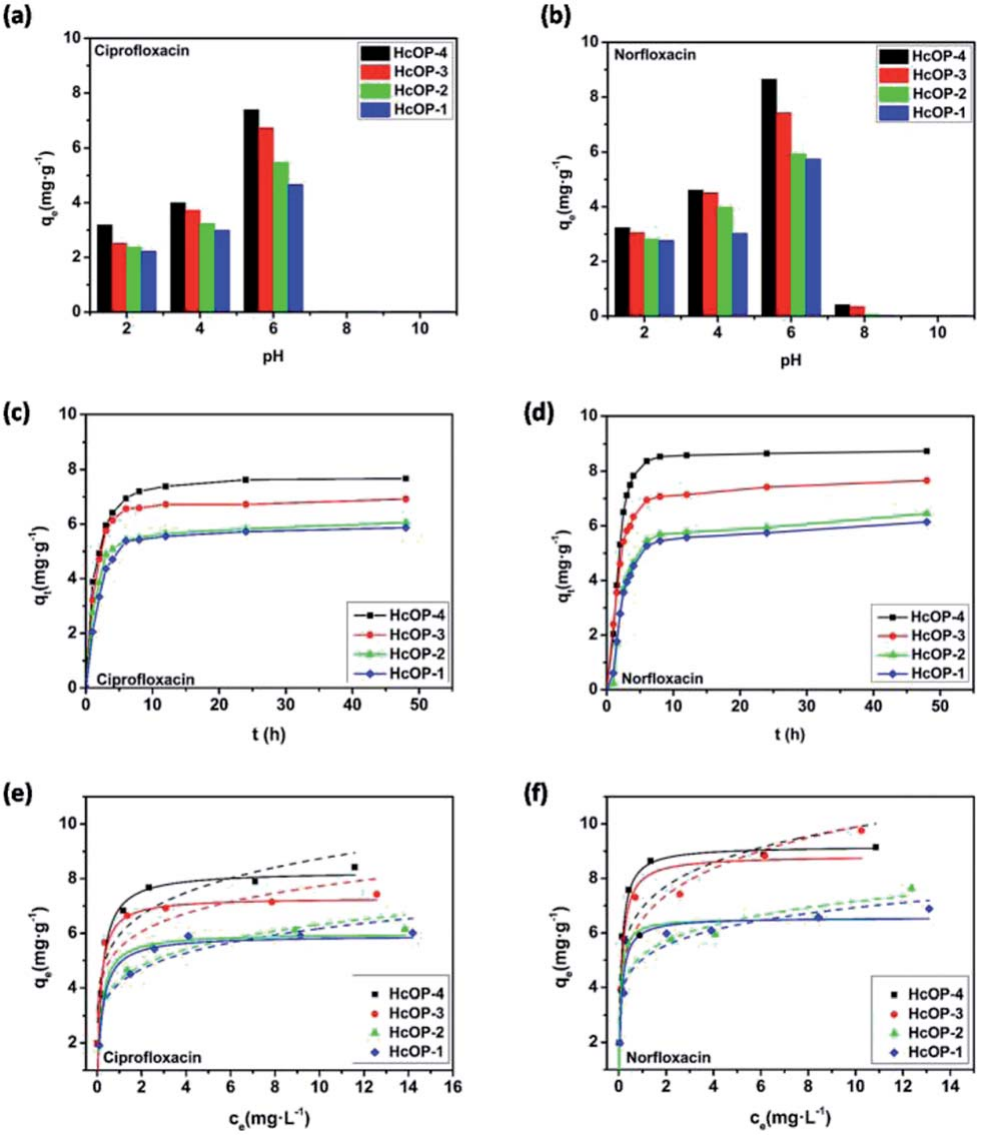
(a) and (b) Effect of solution pH on antibiotics adsorption onto H_C_OPs. (c) and (d) the fluoroquinolone adsorption capacity under different contact times (*C*_0_ = 10 mg L^−1^, pH = 6.0). (e) and (f) Langmuir and Freundlich adsorption isotherms of fluoroquinolones adsorption onto H_C_OPs. Dashed line: Freundlich model; solid line: Langmuir model.

**Fig. 4:**
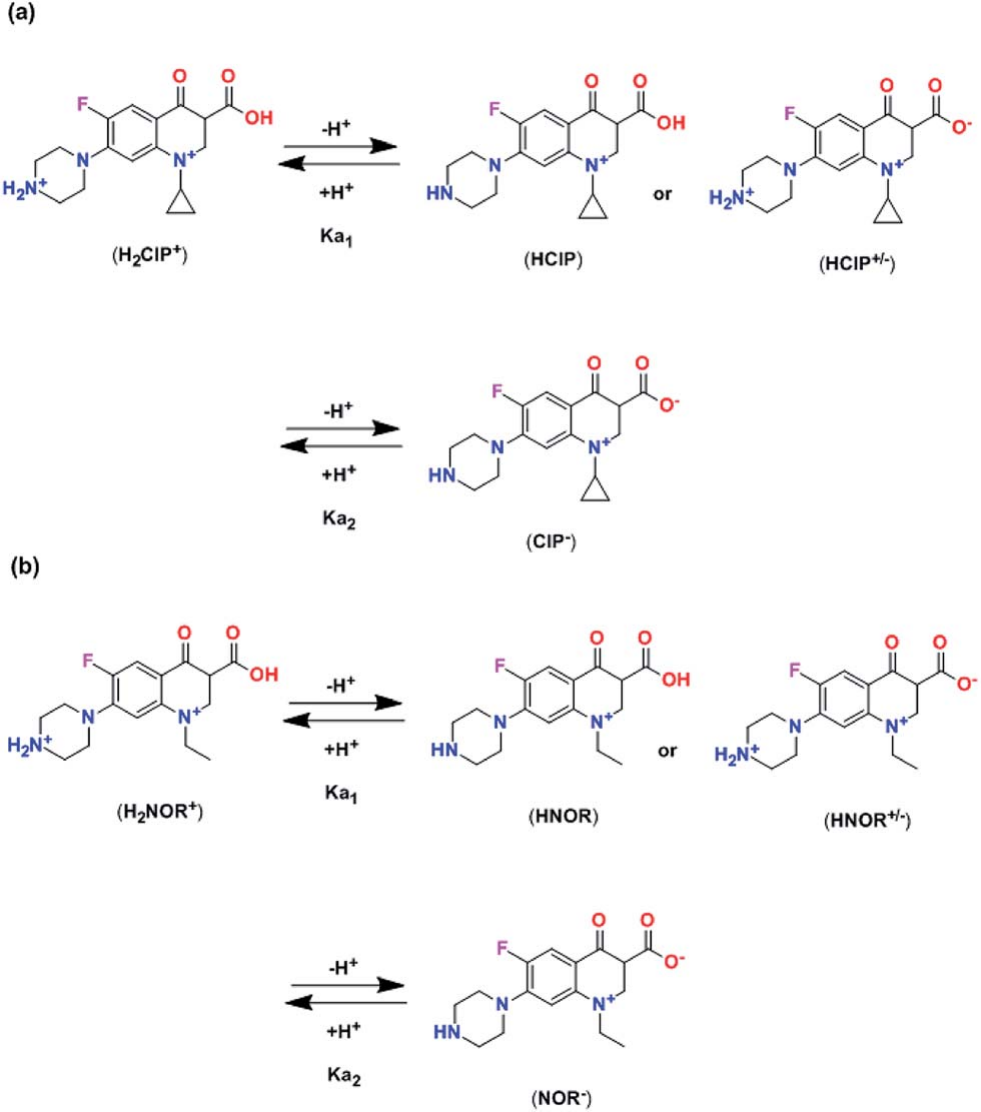
The speciation of process reactive for ciprofloxacin (a) and norfloxacin (b) in aqueous solution as a function of the solution pH.

Adsorption amounts of ciprofloxacin and norfloxacin onto four H_C_OPs at different pH values were depicted in Fig. 3a and b. It can be seen that the equilibrium adsorption capacities (*q*_e_) of ciprofloxacin and norfloxacin both showed the increasing tendency and then decreasing tendency with pH values varied from 2 to 10. This may be related to the various zeta potential values of H_C_OPs and the different species of ciprofloxacin (*K*_a1_ = 6.1, *K*_a2_ = 8.7) and norfloxacin (*K*_a1_ = 6.2, *K*_a2_ = 8.5) changing with pH, to be specific, cationic species (pH < 5.9 ± 0.15), zwitterionic species (6.1 < pH < 8.9) and anionic species (pH > 8.9 ± 0.11).^15^ Typically, ciprofloxacin and norfloxacin contain both N–H and –COOH groups, which can be combined with the H^+^ and OH^−^ in the solution, thus affecting the fluoroquinolones adsorption onto H_C_OPs (Fig. 4). Therefore, the changing of pH induced the mutual transformation of the electrostatic interactions and electrostatic repulsive force between fluoroquinolone molecules and the H_C_OP surfaces, giving rise to the emergence of the maximum adsorption amounts at pH = 6.0. Accordingly, the optimal pH for the four H_C_OPs over the two kinds of fluoroquinolones was pitched on pH = 6.0, which was for studying go a step further.

For the sake of investigating the behaviour of fluoroquinolones onto the H_C_OPs, batch adsorption experiments were conducted at different reaction times for the initial concentration of 10 mg L^−1^ (pH = 6.0). As shown in Fig. 3c and d, the adsorption processes of ciprofloxacin and norfloxacin on H_C_OPs were rapid at the initial adsorption stage of 6 h, after which the adsorption rate no longer changed and eventually entered the adsorption equilibrium process. The adsorption data of the H_C_OPs over fluoroquinolones were processed by pseudo first- and second-order kinetic models, intraparticle diffusion model (Fig. S10, ESI†).^16^ Owing to the higher linear fitting coefficient (*R*^2^) values and closer calculated equilibrium adsorption capacity (*q*_e_,_cal_) values of the two kinetic models, the second-order kinetic model described the nature of adsorption process more suitably (Tables S2 and S4, ESI†). Moreover, the linear relationship between the *q*_*t*_ and *t*0.5 indicated the combined process of intraparticle diffusion and the external mass transfer in adsorption processes of fluoroquinolones onto the H_C_OPs being studied (Tables S3 and S5, ESI†). For the adsorbed rates of antibiotics, the orders are in following sequence of H_C_OP-1 < H_C_OP-2 < H_C_OP-3 < H_C_OP-4, which can be contributed to the differences in specific surface areas and pore sizes.^17^ Besides, the removal efficiencies (*E*) of fluoroquinolones onto the same kind of H_C_OPs increased as *E*_ciprofloxacin_ < *E*_norfloxacin_, which is related to the hydrophobic interactions between the adsorbent and adsorbate.

In order to evaluate the adsorption capacities of fluoroquinolones onto the H_C_OPs, the adsorption amounts were tested as a function of various initial antibiotic concentrations (Fig. 3e and f). Both the Freundlich and Langmuir models were performed to fit test data (Fig. S11, ESI†).^18^ By comparing the linear fitting coefficient (*R*^2^) values of both ciprofloxacin and norfloxacin, we can draw a conclusion that the adsorption process was interpreted reasonably well by the Langmuir isotherm model (Tables S6–S9, ESI†). Based on the data listed, the maximum adsorption capacity (*q*_m_) values calculated from the Langmuir model of four H_C_OPs over two fluoroquinolones were both in the order of H_C_OP-4 > H_C_OP-3 > H_C_OP-2 > H_C_OP-1, which impelled us to believe that the more favorable adsorption over H_C_OP-4 than the adsorption over H_C_OP-3, H_C_OP-2 or H_C_OP-1. This phenomenon is commonly observed in the adsorption process of porous materials, there, greater specific surface area appear to favour the higher adsorption capacity.^19^ The other side, the *q*_m_ values of norfloxacin were larger than that of ciprofloxacin when using the same H_C_OP as adsorbent. It is worth noting that with the values, H_C_OPs could exceed some commercial materials and even some synthetic zeolites, providing the great feasibility as a popular alternative of existing adsorbents which avoids complex synthesis (Table S10†). Afterwards, the morphologies and structures of the H_C_OPs after adsorption were characterized by SEM images and FT-IR analysis in succession. As shown in Fig. S12–16,† the morphologies and structures of fluoroquinolone-loaded networks did not change significantly with regard to the parent networks, which further elaborated on the stability of H_C_OPs.

**Fig. 5:**
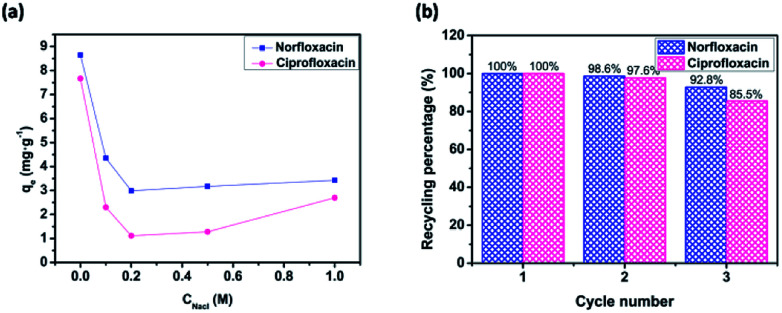
(a) Effect of coexisting ions on adsorption capacity of fluoroquinolones onto H_C_OPs at pH = 6.0. (b) Relative adsorption capacity of fluoroquinolones onto H_C_OPs after recycling.

Since the sodium chloride commonly existed in the most wastewater, the influence of ionic strength (NaCl) on fluoroquinolones adsorption onto cross-sectional H_C_OP-4 was carried out. As shown in Fig. 5a, the decreased adsorption amounts under low NaCl conditions (0 to 0.2 M) may be explained by the competitive adsorption of Na^+^ cations with positive fluoroquinolones on the H_C_OP-4 surface. However, when ionic strength is large enough, the salting out effect between fluoroquinolone molecules and NaCl, which leading to the decreased solubility of fluoroquinolone molecules in aqueous solution, resulting in the enhanced hydrophobic interactions between fluoroquinolones and H_C_OP-4. Therefore, more fluoroquinolones were facilitated to diffuse the surface of the H_C_OP-4, bringing about the increased adsorption amounts of fluoroquinolones under high initial NaCl concentrations (0.5 to 1 M).^20^

The recyclability of the H_C_OP adsorbents towards fluoroquinolones were investigated by using generated adsorbents for the subsequent adsorption cycles under the same conditions to explore the practical value. Similarly, H_C_OP-4 was selected as representative adsorbent for detailed investigation. The results in Fig. 5b demonstrated that the recycling percentage of norfloxacin still maintained 92.8% (while 85.5% for that of ciprofloxacin) of the initial capacity after three recycles, defining the good repeatable application of H_C_OPs in treatment of fluoroquinolones pollution.

### Adsorption mechanism

**Fig. 6:**
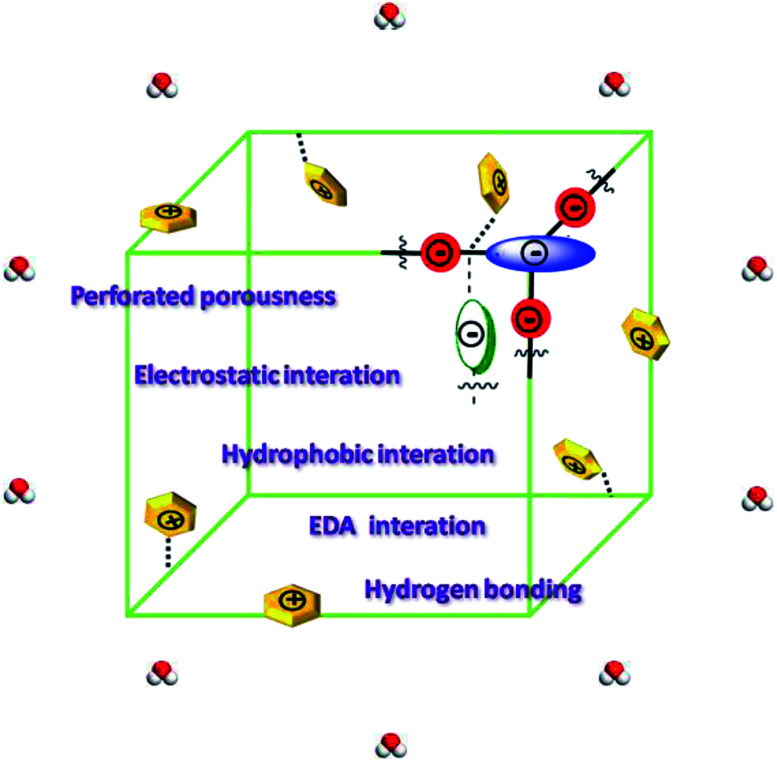
Schematic diagram of possible mechanisms for adsorptive removal of fluoroquinolones.

To get a deep understanding of the mechanisms of adsorption for fluoroquinolones onto the four H_C_OPs, we speculated that it may be attributed to a combination of perforated porousness, electrostatic interaction, hydrophobic interaction, π–π electron-donor–acceptor (EDA) interaction and hydrogen bonding formation (Fig. 6).^21^ Remarkably, the perforated porousness assumed enormous importance in the adsorption progress as discussed before that the large specific surface area is propitious to high adsorption capacity. The electrostatic interaction was without question one of the major effecting factors for antibiotics adsorption onto the H_C_OPs. We are informed of the zeta potential curves that when the pH was at 6.0, the surfaces of all the H_C_OPs were negatively charged (Fig. S8, ESI†); in contrast, the norfloxacin and ciprofloxacin can exist as cationic forms with positive charge. On these grounds, adsorption amounts were enhanced as expected due to the electrostatic attraction force between antibiotic molecules and the H_C_OPs surfaces. Additionally, in terms of higher *E* and *q*_m_ values for norfloxacin in comparison with ciprofloxacin onto H_C_OPs, the dominated role of hydrophobicity in fluoroquinolone adsorption has been fully given weight. Now that norfloxacin and ciprofloxacin could function as π-acceptors on account of electron-withdrawing –F groups; conversely, –CO–NH− functional groups endow the H_C_OPs with π-electron-rich skeletons, which could function as π-donors, the π–π EDA interaction are prone to effect significantly in the fluoroquinolones adsorption onto the H_C_OPs. Last, hydrogen bond information also has a certain impact on the fluoroquinolones adsorption onto the H_C_OPs. The –CO–NH– and –COOH functional groups on H_C_OPs make them could act as hydrogen bond-donors as well as hydrogen bond-acceptors; correspondingly, the norfloxacin and ciprofloxacin could act as both hydrogen bond-acceptors and hydrogen bond-donors due to the –NH, –COOH and –F functional groups.

## Conclusions

In conclusion, four novel porous hydrogen-bonding covalent organic polymers (H_C_OPs) have been developed based on three-composite building blocks as carriers to adsorb emerging fluoroquinolone pollutants *via* a facile method for the first time. The specific surface areas and pore sizes of these porous H_C_OPs, which bringing about the diverse in adsorption capacity, could be regulated to a certain extent by varying the lengths of strut. Or rather, the other three H_C_OPs exhibited comparably low adsorption capacity in contrast to H_C_OP-4, which is a result that the absorbing abilities adhere to the order of porous characteristics. In the meantime, the functional groups on the skeletons of H_C_OPs, such as –CO–NH– and –COOH groups, favored the adsorption of fluoroquinolones. In addition, it can be inferred that other possible controlling mechanisms such as electrostatic interaction, hydrophobic interaction, π–π electron-donor–acceptor (EDA) interaction and hydrogen bonding formation also accompanied with effect on the fluoroquinolones adsorption onto H_C_OPs.

## Conflicts of interest

There are no conflicts to declare.

## Supplementary Material

RA-008-C8RA06806B-s001
