# Supplementary material for: Facile synthesis of novel porous self-assembling hydrogen-bonding covalent organic polymers and their applications towards fluoroquinolone antibiotics adsorption
Source: RSC Adv. 2018 Oct 1;8(58):33516–22. doi: 10.1039/c8ra06806b (PMC9086438; doi:10.1039/c8ra06806b)
Supplement: RA-008-C8RA06806B-s001 [file RA-008-C8RA06806B-s001.pdf]

Electronic Supplementary Material (ESI) for RSC Advances.

This journal is © The Royal Society of Chemistry 2018

Supporting information for

Facile synthesis of novel porous self-assembling hydrogen-bonding covalent organic polymers and their applications towards fluoroquinolone antibiotics adsorption

Zhuoran Li,[a] Feifan Xu,[a] Zhi Liu,[b] Chuanyu Qin,[a] Hao Ren[c] and Yangxue Li[a*]

[a.] *Key Lab of Groundwater Resources and Environment, Ministry of Education, Jilin University, 2519 Jiefang Road, Changchun 130021, P. R. China;*

[b.] *School of Municipal and Environmental Engineering, Jilin Jianzhu University, 5088 Xincheng Street, Changchun 130118, P. R. China;*

[c.] *State Key Laboratory of Inorganic Synthesis and Preparative Chemistry, College of Chemistry, Jilin University, Changchun 130012, P.R. China*

1. PXRD spectra
2. FT-IR spectra
3. TGA curves
4. N_2 adsorption isotherms and pore size distribution
5. Zeta potential curves
6. Adsorption experiments
7. Tables
8. SEM images of the H_COPs after fluoroquinolones adsorption
9. FT-IR spectra of the H_COPs after fluoroquinolones adsorption
10. Supporting references

1. PXRD spectra

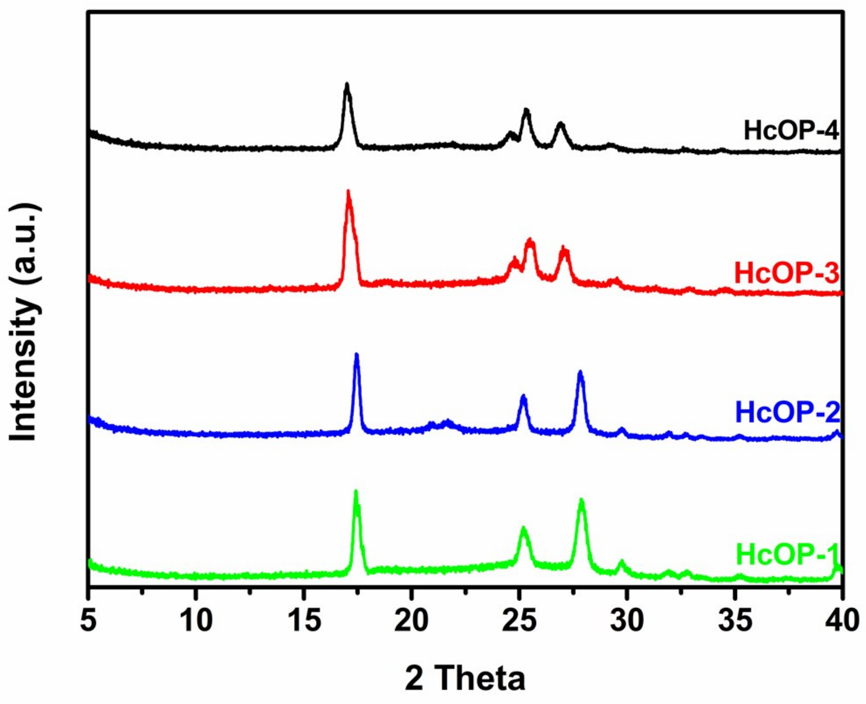

Figure S1 PXRD spectra of H_COPs.

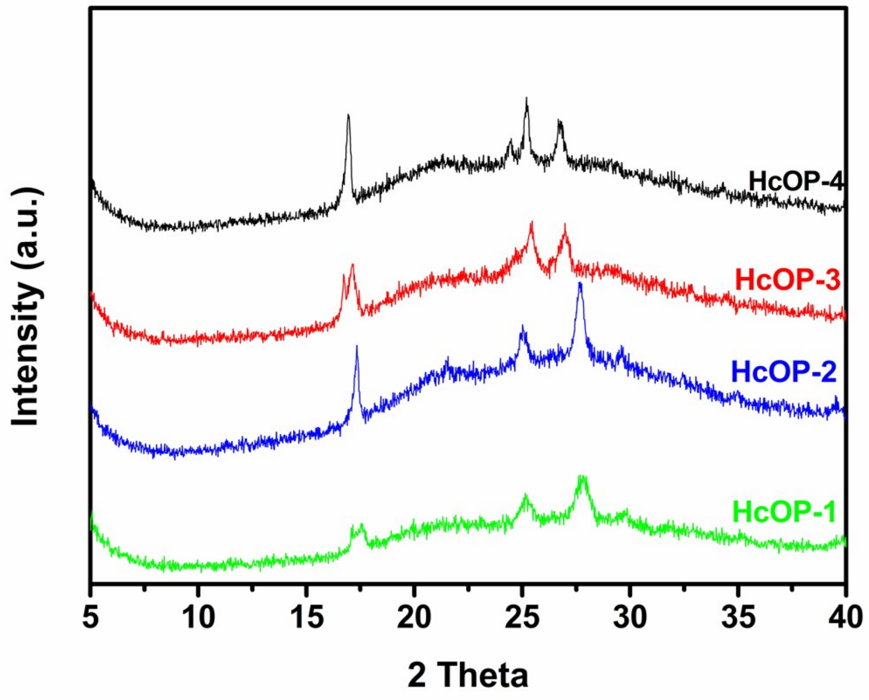

Figure S2 PXRD spectra of H_COPs after soaking in concentrated HCl for 3 days.

2. FT-IR spectra

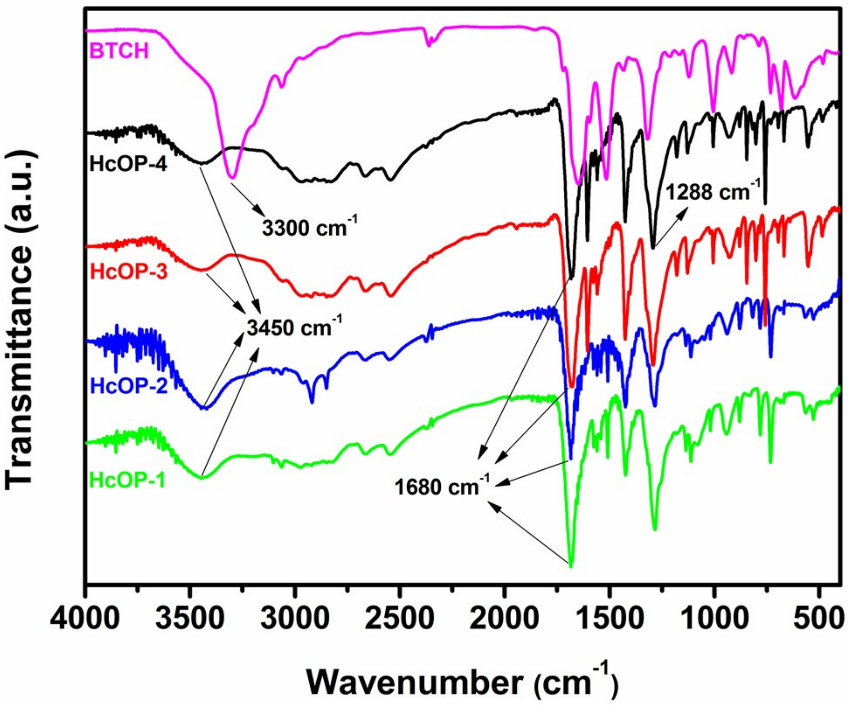

Figure S3 FT-IR spectra of BTCH monomer and H_COPs.

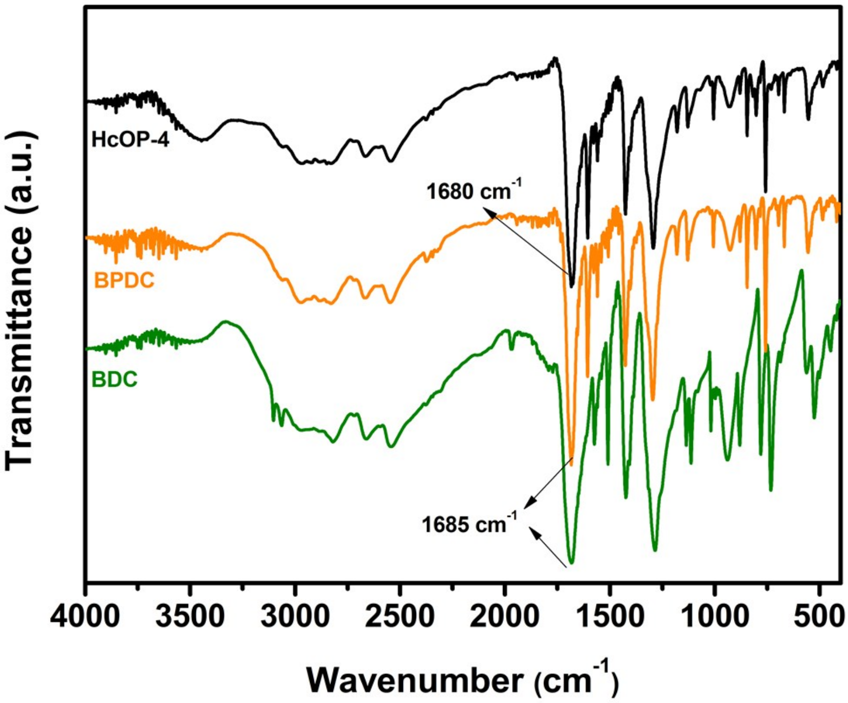

Figure S4 FT-IR spectra of BDC (or BPDC) monomers and H_COP-4.

3. TGA curves

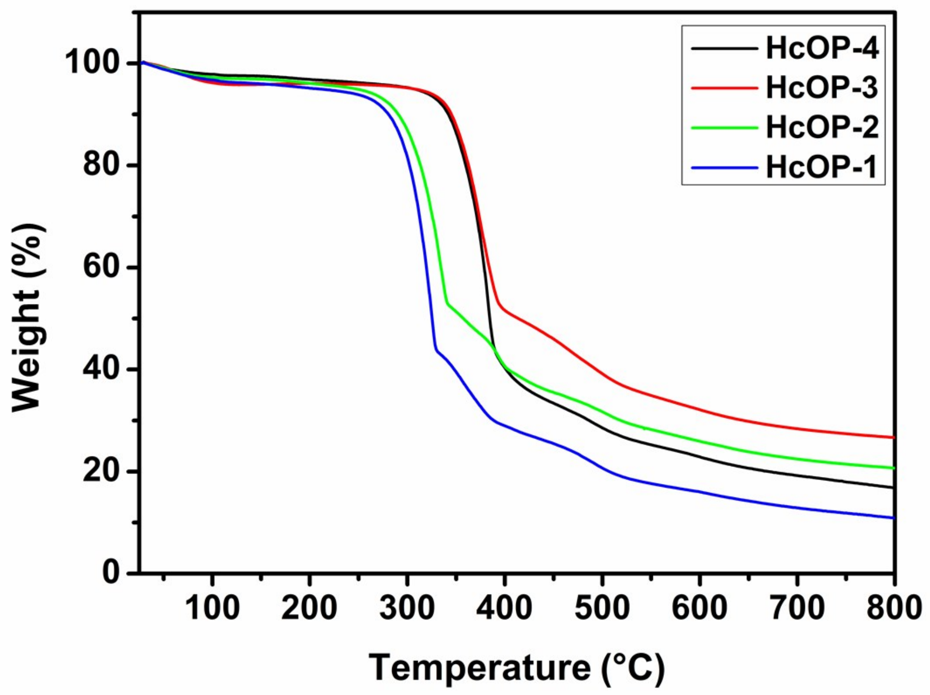

Figure S5 TGA curves of H_COPs.

4. N_2 adsorption isotherms and pore size distribution

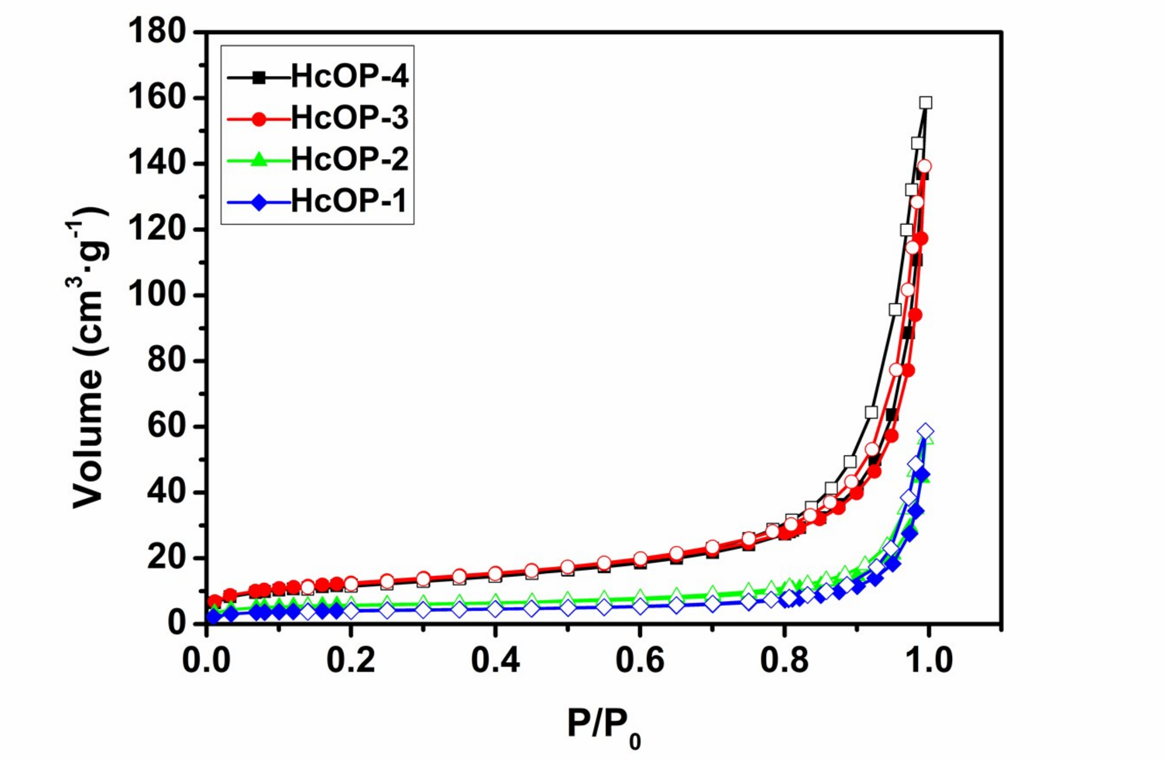

Figure S6 Nitrogen adsorption and desorption isotherms of H_COPs measured at 77 K.

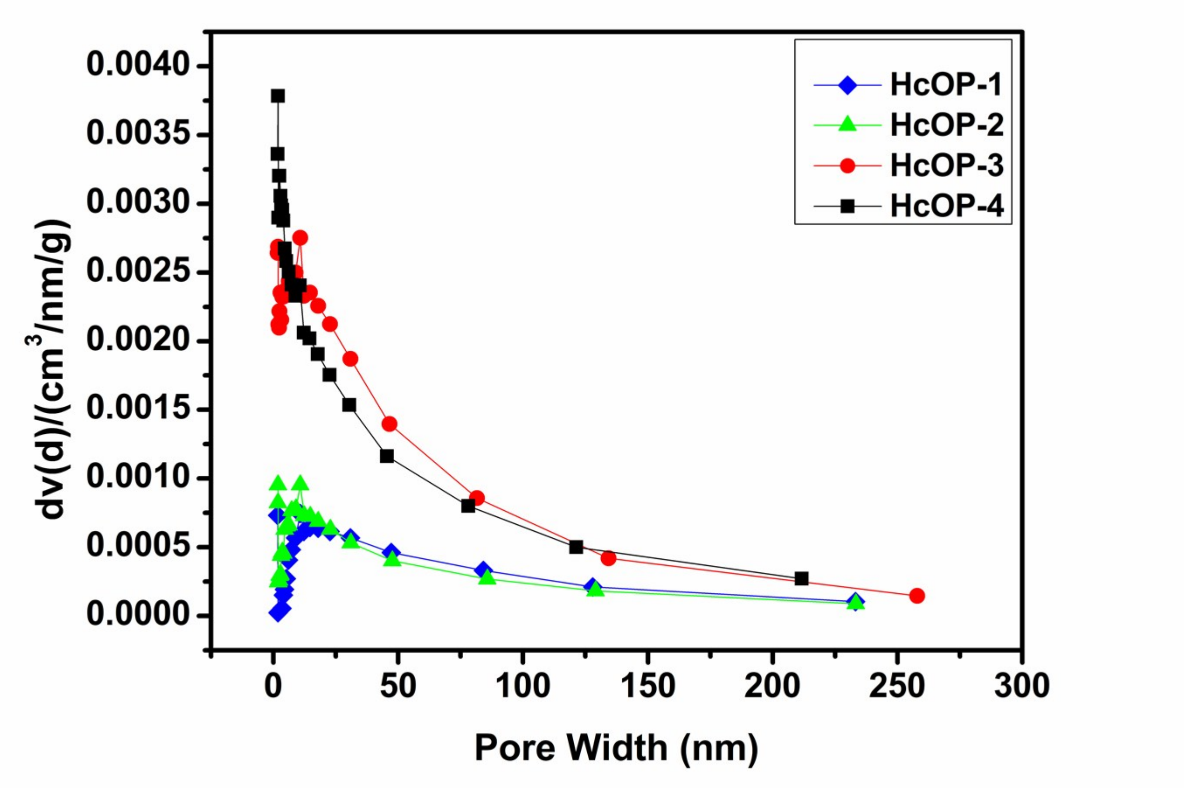

Figure S7 Pore size distributions of H_COPs.

Table S1 Adsorption characteristics of H_COPs.

H_COPs	*BET* *(m^2 g^{-1})*	*Langmuir* *(m^2 g^{-1})*	*Pore volume* *(cm^3 g^{-1})*
**H_COP-1**	**13**	**20**	**0.04**
**H_COP-2**	**18**	**28**	**0.04**
**H_COP-3**	**41**	**65**	**0.12**
**H_COP-4**	**43**	**68**	**0.14**

5. Zeta potential curves

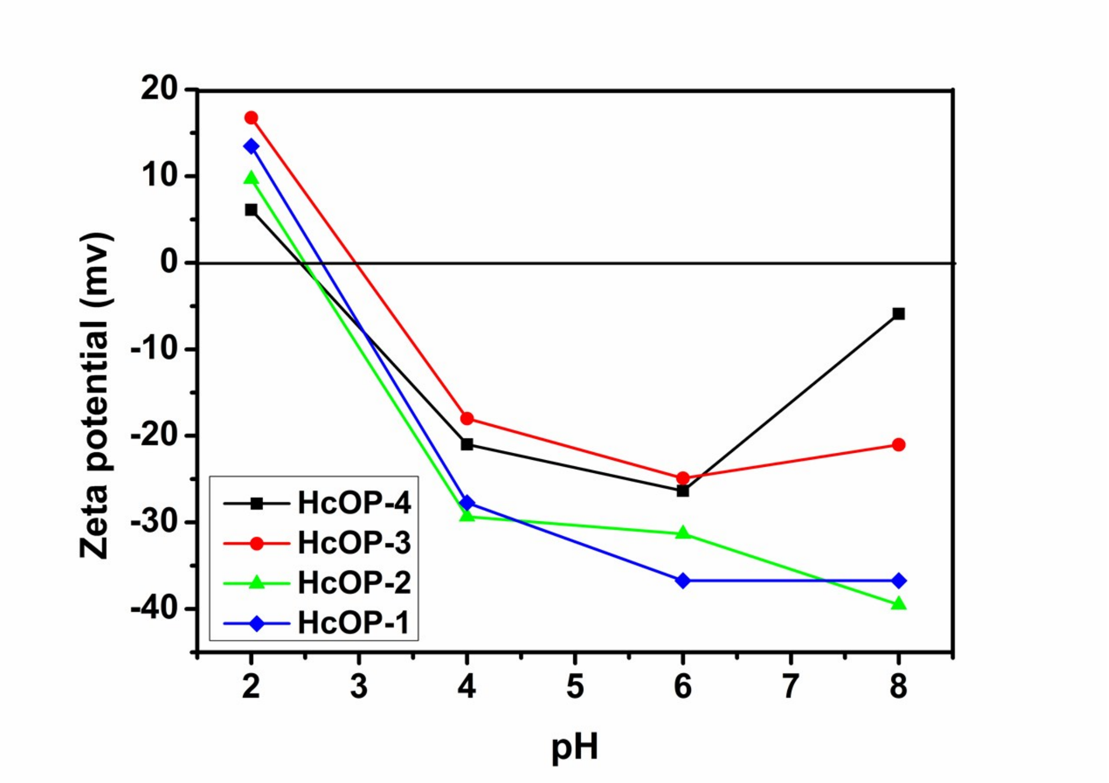

Figure S8 Zeta potential curves *versus* pH of the H_COPs.

6. Adsorption experiments

Equations 1, 2 and 3 were used to calculate the removal efficiency (E, %), the adsorption amount of fluoroquinolones by H_COPs at desired time t (q_t, mg g^{-1}) and the adsorption capacity of fluoroquinolones by H_COPs at equilibrium (q_e, mg g^{-1}), respectively:[1]

$$E(\%) = \frac{C_0 - C_e}{C_0} \times 100\% \tag{1}$$

$$q_e = \frac{(C_0 - C_e)V}{m} \tag{2}$$

$$q_t = \frac{(C_0 - C_t)V}{m} \tag{3}$$

where C_0 (mg L^{-1}) and C_e (mg L^{-1}) are the initial concentrations of fluoroquinolones and the equilibrium concentrations of fluoroquinolones, respectively; m (g) is the mass of adsorbent used; V (L) is the total volume of solution used.

6.1 Calibration plots of standard ciprofloxacin and norfloxacin

A serious of fluoroquinolone solutions with different concentrations at a range of pH values from 2.0 to 10.0 were prepared as standards. The calibrated plots all exhibited a good correlation coefficient.

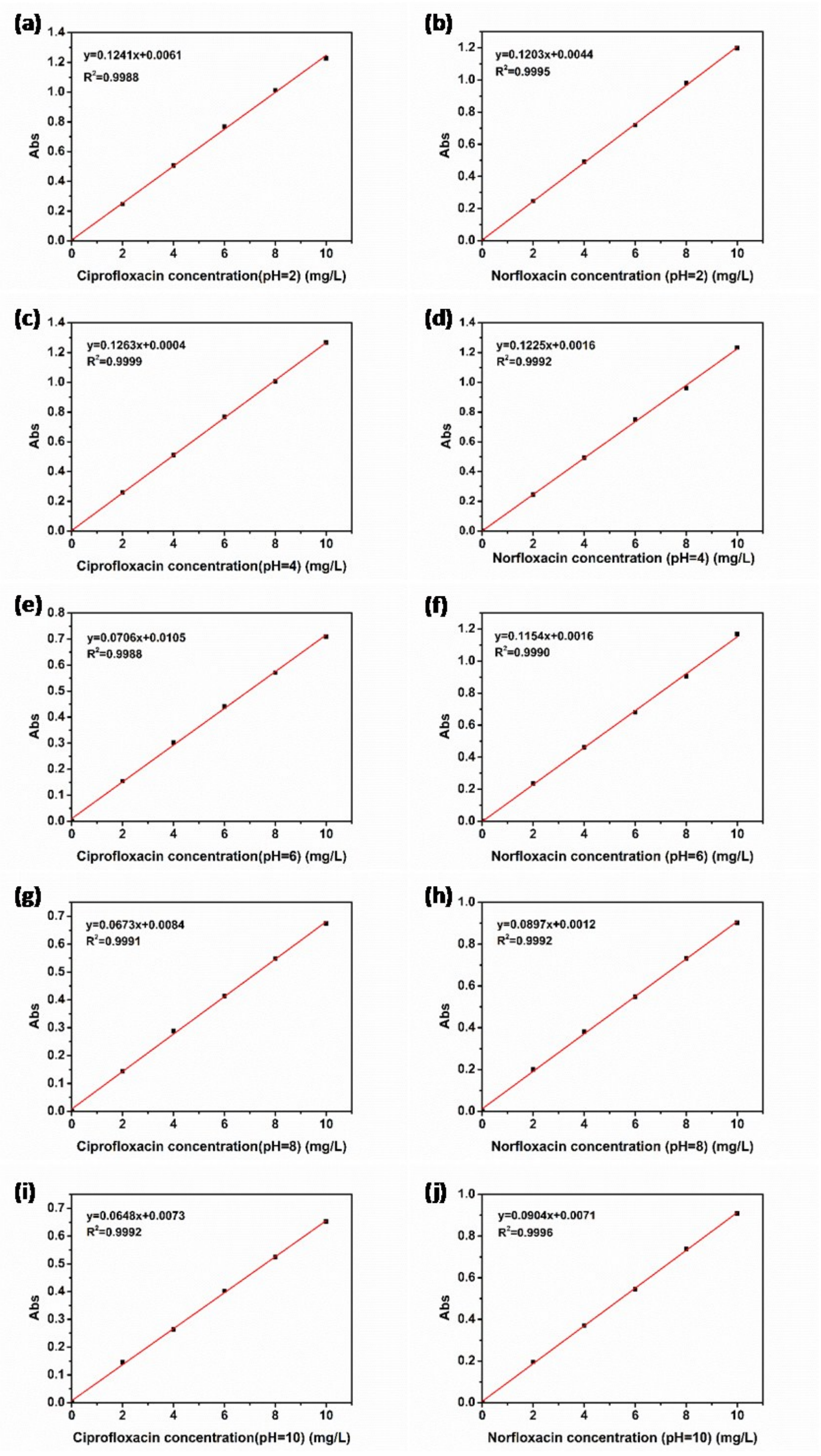

Figure S9 The standard curves of the ciprofloxacin and norfloxacin.

6.2 Adsorption kinetic

The adsorption kinetic were studied using experiments with different contacting time at initial fluoroquinolone concentration of 10 mg/L at pH = 6.0. Pseudo-first-order kinetic, pseudo-second-order kinetic models and intraparticle diffusion model are described as the following functions:[2–4]

$$\ln\left(q_e - q_t\right) = \ln q_e - k_1 t \tag{4}$$

$$\frac{t}{q_t} = \frac{1}{k_2 q_e^2} + \frac{t}{q_e} \tag{5}$$

$$q_t = k_i t^{1/2} + C \tag{6}$$

where q_t (mg g^{-1}) and q_e (mg g^{-1}) represent the same as above; k_1 (h^{-1}) and k_2 (g mg^{-1} h^{-1}) are the pseudo-first-order and the pseudo-second-order rate constants, respectively. k_i (mg g^{-1} h$^{-1/2}$) is the diffusion rate constant and C (mg g^{-1}) is the intercept which are proportional to the extent of boundary layer thickness.

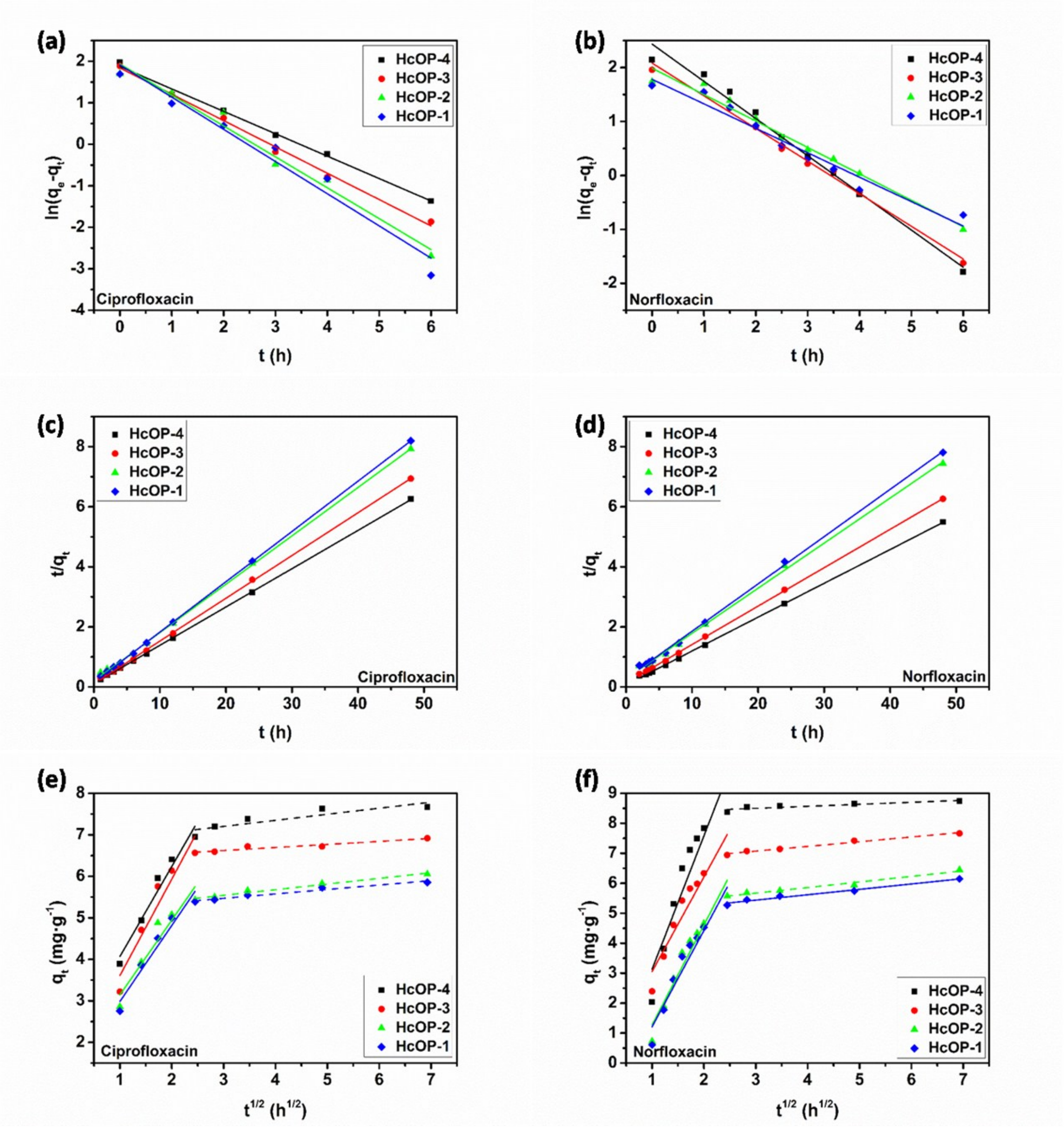

Figure S10 (a) and (b) Pseudo-first-order curve-fittings for fluoroquinolones adsorption onto H_COPs; (c) and (d) Pseudo-second-order curve-fittings for fluoroquinolones adsorption onto H_COPs; (e) and (f) Intraparticle diffusion models for fluoroquinolones adsorption onto H_COPs.

6.3 Adsorption isotherms analysis

The Langmuir sorption isotherm is often applied for the monolayer adsorption process, while the well-known Freundlich isotherm is based on sorption on heterogeneous surface with multilayer adsorption. The Langmuir isotherm and Freundlich isotherm models can be represented as following equations:[5–6]

$$\frac{C_e}{q_e} = \frac{1}{bq_m} + \frac{C_e}{q_m} \quad (7)$$

$$\ln q_e = \ln K_F + \frac{1}{n}\ln C_e \quad (8)$$

where q_e (mg L^{-1}) is the adsorption capacity at equilibrium status, C_e (mg L^{-1}) is the concentration of fluoroquinolones in solution at equilibrium status, and b (L mg^{-1}) is the Langmuir constant. In addition, the q_m is the maximum adsorption capacity of the adsorbent. K_F (L g^{-1}) is the Freundlich constant concerned with the adsorption capacity and $1/n$ corresponds to the heterogeneity factor.

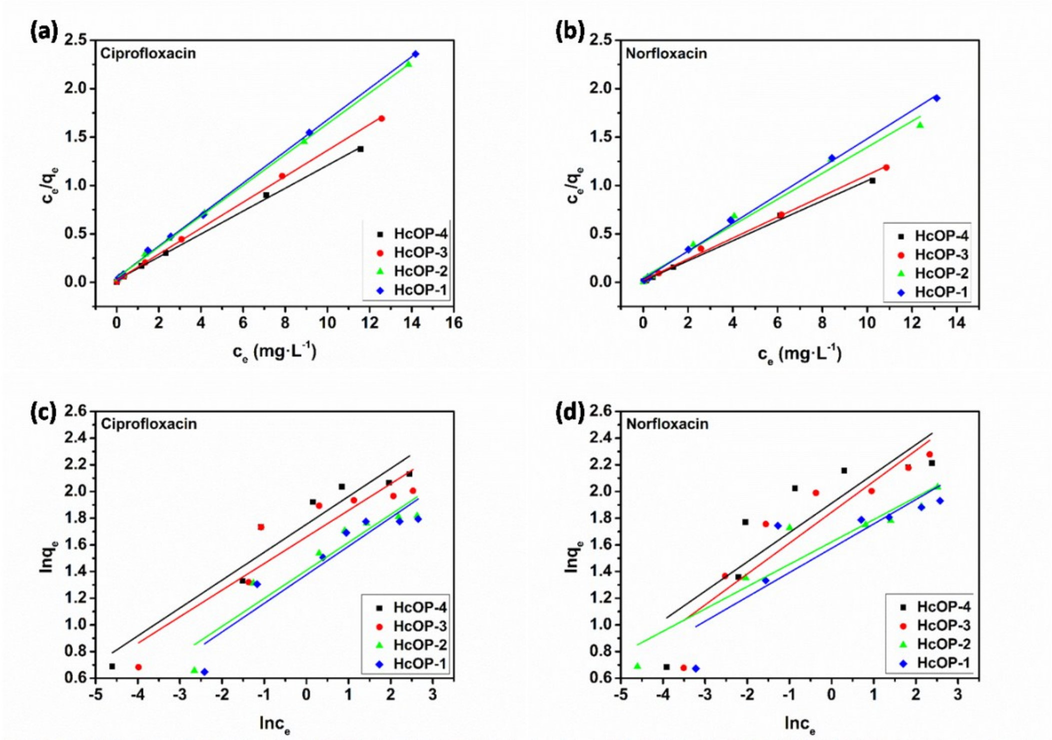

Figure S11 (a) and (b) Langmuir linear fittings for the fluoroquinolones adsorption onto H_COPs; (c) and (d) Freundlich linear fittings for the fluoroquinolones adsorption onto H_COPs .

7. Tables

Table S2 Removal efficiencies and kinetic parameters for the adsorption of ciprofloxacin onto H_COPs.

H_COPs	*Removal Efficiency (%)*	$q_{e,exp}$ ($mg \cdot g^{-1}$)	Pseudo-first-order kinetics			Pseudo-second-order kinetics		
			k_1 (h^{-1})	$q_{e,cal}$ ($mg \cdot g^{-1}$)	R^2	k_2 ($g \cdot mg^{-1} \cdot h^{-1}$)	$q_{e,cal}$ ($mg \cdot g^{-1}$)	R^2
**H_COP-1**	**57.3%**	**5.73**	**0.78**	**6.30**	**0.96**	**0.13**	**5.97**	**0.99**
**H_COP-2**	**58.3%**	**5.83**	**0.75**	**6.55**	**0.98**	**0.14**	**6.21**	**0.99**
**H_COP-3**	**67.2%**	**6.72**	**0.64**	**6.59**	**0.98**	**0.18**	**6.80**	**0.99**
**H_COP-4**	**76.3%**	**7.63**	**0.54**	**6.89**	**0.98**	**0.19**	**7.84**	**0.99**

Table S3 Intraparticle diffusion model parameters for the adsorption of ciprofloxacin onto H_COPs.

H_COPs	Intraparticle diffusion model					
	$k_{i,1}$ ($mg \cdot g^{-1} \cdot h^{-1/2}$)	C_1 ($mg \cdot g^{-1}$)	R^2	$k_{i,2}$ ($mg \cdot g^{-1} \cdot h^{-1/2}$)	C_2 ($mg \cdot g^{-1}$)	R^2
**H_COP-1**	**1.83**	**1.15**	**0.94**	**0.11**	**5.13**	**0.95**
**H_COP-2**	**1.80**	**1.35**	**0.88**	**0.14**	**5.13**	**0.94**
**H_COP-3**	**2.32**	**1.29**	**0.89**	**0.07**	**6.40**	**0.89**
**H_COP-4**	**2.17**	**1.90**	**0.95**	**0.15**	**6.77**	**0.72**

Table S4 Removal efficiencies and kinetic parameters for the adsorption of norfloxacin onto H_COPs.

H_COPs	*Removal Efficiency (%)*	$q_{e,exp}$ $(mg \cdot g^{-1})$	Pseudo-first-order kinetics			Pseudo-second-order kinetics		
			k_1 (h^{-1})	$q_{e,cal}$ $(mg \cdot g^{-1})$	R^2	k_2 $(g \cdot mg^{-1} \cdot h^{-1})$	$q_{e,cal}$ $(mg \cdot g^{-1})$	R^2
**H_COP-1**	**58.6%**	**5.86**	**0.45**	**5.03**	**0.96**	**0.08**	**6.35**	**0.99**
**H_COP-2**	**60.6%**	**6.06**	**0.49**	**7.32**	**0.98**	**0.09**	**6.66**	**0.99**
**H_COP-3**	**69.2%**	**6.92**	**0.61**	**8.01**	**0.98**	**0.12**	**7.82**	**0.99**
**H_COP-4**	**76.7%**	**7.67**	**0.69**	**11.35**	**0.98**	**0.18**	**8.87**	**0.99**

Table S5 Intraparticle diffusion model parameters for the norfloxacin of ciprofloxacin onto H_COPs.

H_COPs	Intraparticle diffusion model					
	$k_{i,1}$ $(mg \cdot g^{-1} \cdot h^{-1/2})$	C_1 $(mg \cdot g^{-1})$	R^2	$k_{i,2}$ $(mg \cdot g^{-1} \cdot h^{-1/2})$	C_2 $(mg \cdot g^{-1})$	R^2
**H_COP-1**	**3.21**	**2.00**	**0.91**	**0.18**	**4.90**	**0.97**
**H_COP-2**	**3.33**	**2.06**	**0.93**	**0.19**	**5.12**	**0.97**
**H_COP-3**	**3.15**	**1.10**	**0.89**	**0.16**	**6.60**	**0.98**
**H_COP-4**	**4.45**	**1.32**	**0.85**	**0.07**	**8.30**	**0.74**

Table S6 Adsorption parameters of Langmuir adsorption isotherm model for the adsorption of ciprofloxacin onto H_COPs.

Langmuir isotherm	H_COPs	q_m	K_L	R^2
L	H_COP-1	6.13	3.44	0.99
	H_COP-2	6.29	3.46	0.99
	H_COP-3	7.46	5.76	0.99
	H_COP-4	8.47	4.60	0.99

Table S7 Adsorption parameters of Freundlich adsorption isotherm model for the adsorption of ciprofloxacin onto H_COPs.

Freundlich isotherm	H_COPs	n	K_F	R^2
F	H_COP-1	4.67	3.96	0.85
	H_COP-2	4.77	4.09	0.88
	H_COP-3	5.02	5.26	0.84
	H_COP-4	4.78	5.78	0.91

Table S8 Adsorption parameters of Langmuir adsorption isotherm model for the adsorption of norfloxacin onto H_COPs.

Langmuir isotherm	H_COPs	q_m	K_L	R^2
L	H_COP-1	6.89	4.44	0.99
	H_COP-2	7.44	2.62	0.99
	H_COP-3	9.22	4.62	0.99
	H_COP-4	9.67	6.55	0.99

Table S9 Adsorption parameters of Freundlich adsorption isotherm model for the adsorption of norfloxacin onto H_COPs.

Freundlich isotherm	H_COPs	n	K_F	R^2
F	H_COP-1	5.48	4.83	0.72
	H_COP-2	5.97	5.06	0.88
	H_COP-3	4.32	6.33	0.82
	H_COP-4	4.55	6.77	0.75

Table S10 Comparison of the maximum adsorption capacity of fluoroquinolones on different adsorbents.

S.N.	Adsorbents	Pollutants	Adsorption capacity (mg g^{-1})	T (K)	Reference
1	Modified coal fly ash	CIP	1.55	313	7
2	Kaolinite	CIP	3.26	295	7
3	Kaolinite	CIP	7.95	308	8
4	MCM-41	NOR	1.74	298	9
5	C_{16}-MCM-41	NOR	1.52	298	9
**6**	**HcOP-1**	**CIP / NOR**	**6.13 / 6.89**	**298**	**This work**
**7**	**HcOP-2**	**CIP / NOR**	**6.29 / 7.44**	**298**	**This work**
**8**	**HcOP-3**	**CIP / NOR**	**7.46 / 9.22**	**298**	**This work**
**9**	**HcOP-4**	**CIP / NOR**	**8.47 / 9.67**	**298**	**This work**

8. SEM spectra of the H_COPs after fluoroquinolones adsorption

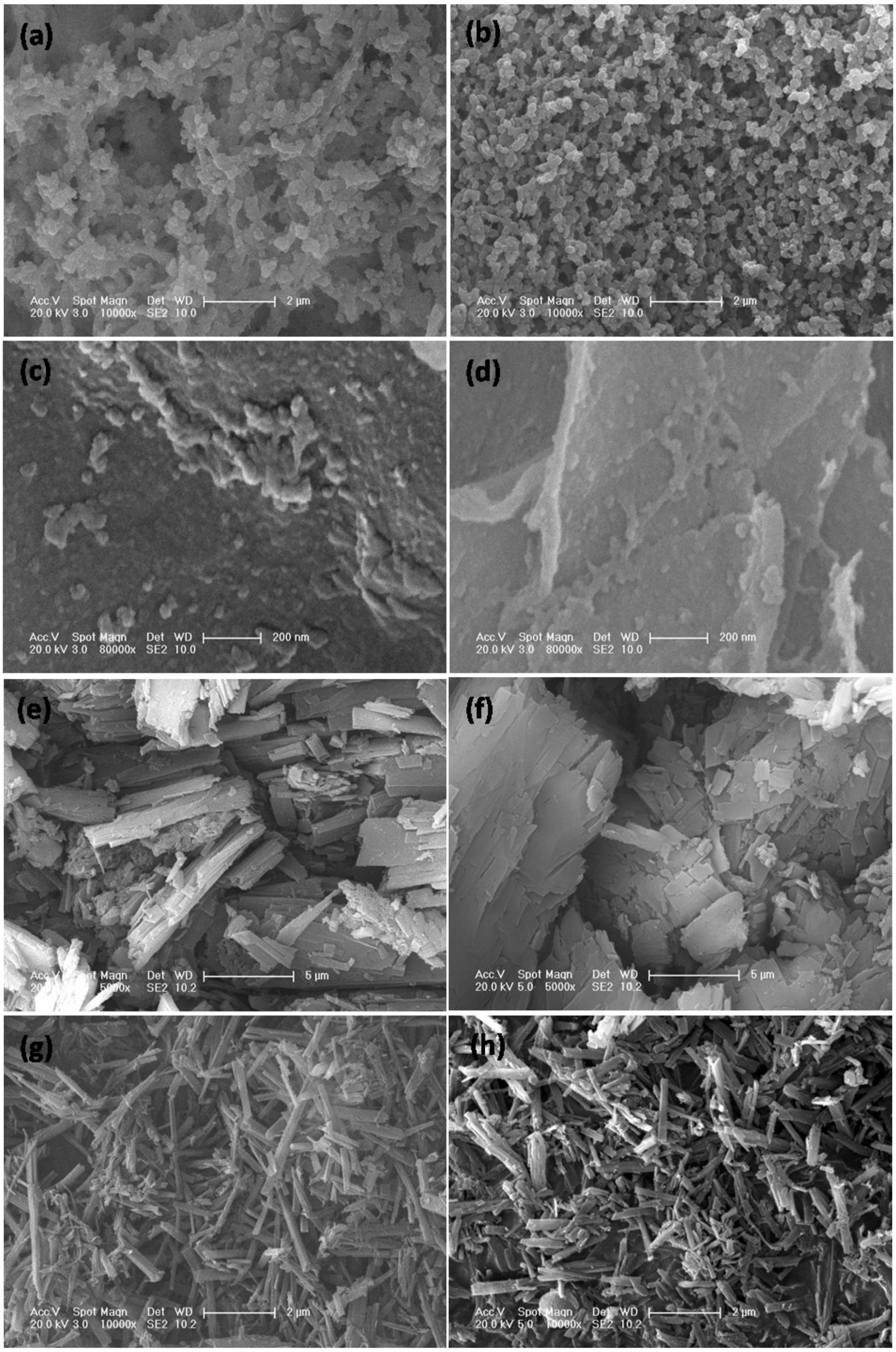

Figure S12 SEM images of (a) CIP@H_COP-1, (b) NOR@H_COP-1, (c) CIP@H_COP-2, (d) NOR@H_COP-2, (e) CIP@H_COP-3, (f) NOR@H_COP-3, (g) CIP@H_COP-4 and (h)

NOR@H$_C$OP-4.

9. FT-IR spectra of the H$_C$OPs after fluoroquinolones adsorption

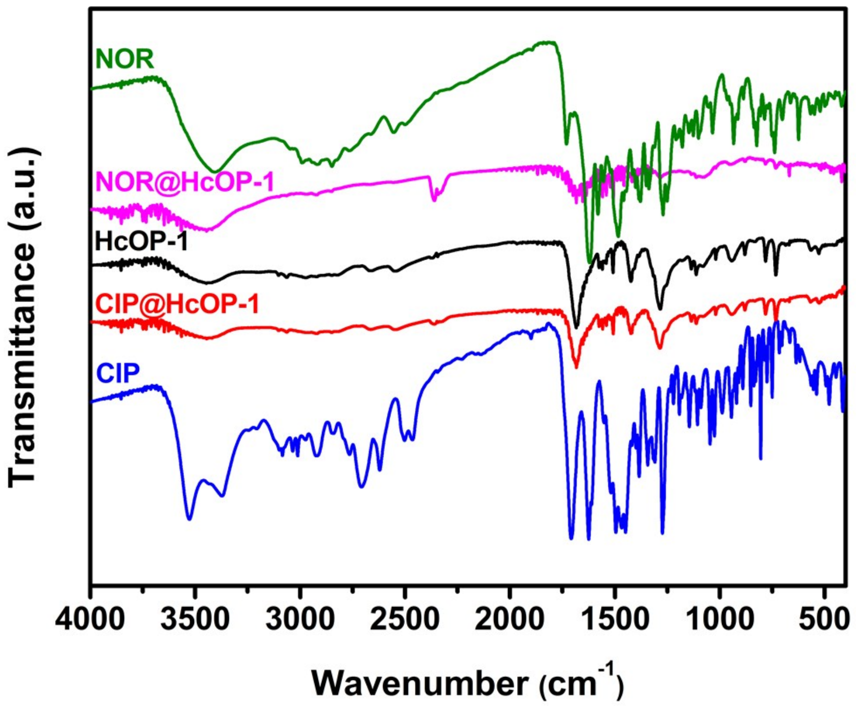

Figure S13 FT-IR spectra of CIP, NOR, H$_C$OP-1, CIP@H$_C$OP-1 and NOR@H$_C$OP-1.

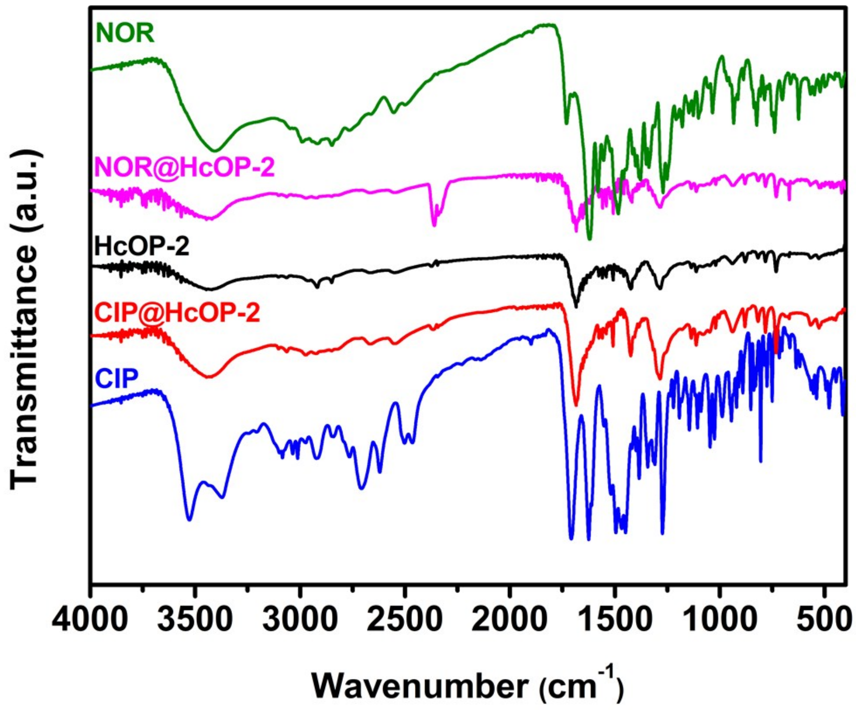

Figure S14 FT-IR spectra of CIP, NOR, H_COP-2, CIP@H_COP-2 and NOR@H_COP-2.

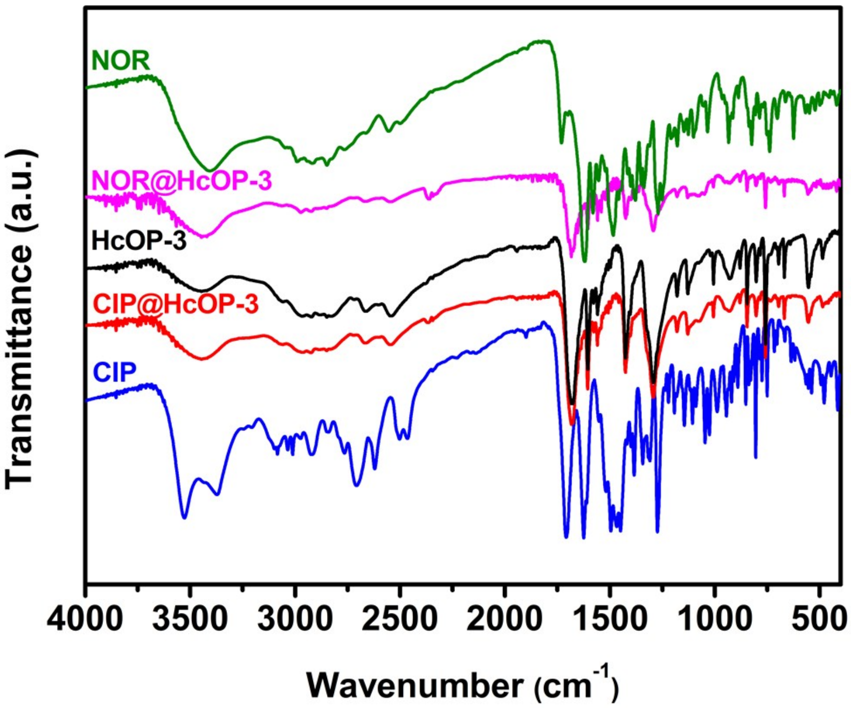

Figure S15 FT-IR spectra of CIP, NOR, H_COP-3, CIP@H_COP-3 and NOR@H_COP-3.

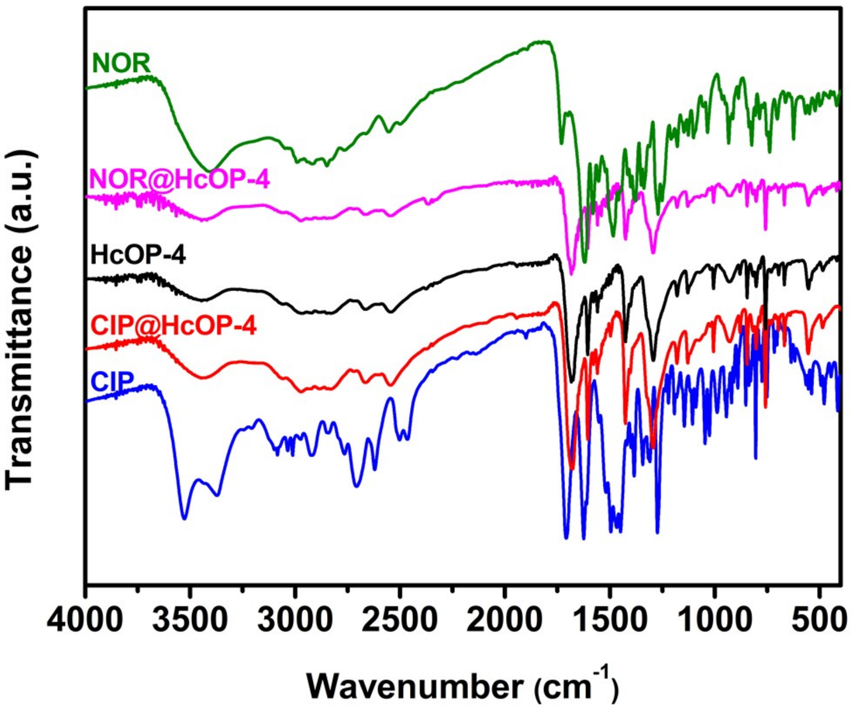

Figure S16 FT-IR spectra of CIP, NOR, H_COP-4, CIP@H_COP-4 and NOR@H_COP-4.

10. Supporting references

[1] J. M. Duan and B. Su, *Chem. Eng. J.*, 2014, **246**, 160–167.

[2]S. Lagergren, Kungliga Svenska Vetenskapsakademiens Handlingar, 1898, 24, 1−39.

[3]Y. S. Ho and G. McKay, *Process Biochem.*, 1999, **34**, 451–465.

[4] S. X. Duan, R. F. Tang, Z. C. Xue, X. X. Zhang, Y. Y. Zhao, W. Zhang, J. H. Zhang, B. Q. Wang, S. Y. Zeng and D. Z. Sun, *Colloids Surf., A*, 2015, **469**, 211−223.

[5] I. Langmuir, *J. Am. Chem. Soc.*, 1918, **40**, 1361–1403.

[6] H. Freundlich and W. Heller, *J. Am. Chem. Soc.*, 1939, **61**, 2228–2230.

[7] X. M. Peng, F. P. Hu, F. L. Y Lam, Y. J. Wang, Z. M. Liu and H. L. Dai, *J. Colloid Interface Sci.*, 2015, **460**, 349–360.

[8] A. A. Mackay and D. E. Seremet, *Environ. Sci. Technol.*, 2008, **42**, 8270–8276.

[9] Z. J. Liang, Z. W. Zhao, T. Y. Sun, W. X. Shi and F. Y. Cui, *J. Hazard. Mater.*, 2016, **305**, 8–14.
